# A set of MATLAB routines and associated files for prediction of radiation-enhanced diffusion in ion irradiated materials

**DOI:** 10.1016/j.dib.2018.09.124

**Published:** 2018-10-04

**Authors:** Peter J. Doyle, Kelsa M. Benensky, Steven J. Zinkle

**Affiliations:** a Department of Nuclear Engineering, University of Tennessee, Knoxville, TN 37996, USA; b Oak Ridge National Laboratory, Oak Ridge, TN 33831, USA

## Abstract

This article presents MATLAB routines that may be used to evaluate radiation-enhanced diffusion (RED) in ion irradiation materials. Four routines are included: Main, DataCollect, Diffuse, and Directory. A sample input file and README are also included. The input may be directly modified as provided and used as an input to the routines. Data from Stopping Range of Ions in Matter (SRIM) is also required as an input. A stream of data files at different damage conditions is created by the routines.

**Specifications table**

**Table:** 

Subject area	*Materials Science*
More specific subject area	*Ion Irradiation of Materials*
Type of data	*Text files (.txt) and MATLAB Routines (.m)*
How data was acquired	*Computer simulation*
Data format	*Simple text*
Experimental factors	*SRIM data is required. Inputs are described below.*
Experimental features	*N/A*
Data source location	*N/A*
Data accessibility	*Accessible through this article or by email of corresponding author*

**Value of the data**

• Output ion profiles may be used to determine appropriate settings of planned ion irradiations
• Iterative use of this program to match experimental implantation profiles to simulated profiles will provide accurate diffusion coefficients and damage rate scaling factors for a given material system
• Experimental diffusion coefficients may then be broadly applied to validate data from previously-performed irradiations

## 1 Data

The routines output data files containing implanted ion atomic fractions, atomic fractions per dpa, locations where implanted ion atomic fractions and atomic fractions per damage surpass supplied thresholds, and damage level across a given material depth. Data files are created in increments specified by the user, a header file containing inputs to the routine is also created with a plot of the implanted ion and damage profiles at the final damage condition compared to the SRIM predictions. Files are output in MATLAB data (.mat) and simple text (.txt) format.

## 2 Experimental design, materials and methods

A full description of the routine development is given in the accompanying article [1]. The Stopping Range of Ions in Matter (SRIM 2008) program [2] is used with parameters as recommended in Stoller et al. [3]. Outputs from this program, COLLISON.txt and RANGE_3D.txt, are read into the routine. Created vacancies and implanted ion locations are then binned as determined by the user. Thermal and peak diffusion coefficients are provided by the user and a total diffusion in each bin is calculated with Eq. (1). $D_{i}$ and $D_{\max}$ are the RED diffusion coefficients of bin “i” and the bin with the maximum damage rate, respectively, $RD_{i}$ and $RD_{\max}$ are the damage rates in bin “i” and at the maximum damage rate, E is the exponential scaling factor which varies between 1.0 and 0.5 for sink-dominant and recombination-dominant conditions, respectively [4], [5], and $D_{\mathrm{therm}}$ is the thermal diffusion coefficient. The first bin׳s diffusion coefficient is set to D_therm_ to simulate a surface sink [4], [5], [6], [7].

(1) $$D_{i}=D_{\max}{\left(\frac{RD_{i}}{RD_{\max}}\right)}^{E}+D_{\mathrm{therm}}$$

Ions are added to each bin in incremental time steps, dt, and then allowed to diffuse over that time step using Fick׳s second law. Following discretization, the change in flux across a bin is found by Eq. (2), where the subscripts indicate the bin under consideration, “i”, and the adjacent bins, “i+1” and “i-1”, Δt is the time step, Δx is the size of the bin, C stands for implanted ion concentration, and D is the bin׳s diffusion coefficient.

(2) $$∴\Delta C_{i}=\frac{\Delta t}{\Delta x^{2}}\left[\frac{\left(D_{i+1}-D_{i-1}\right)\left(C_{i+1}-C_{i-1}\right)}{4}+D_{i}\left(C_{i+1}-2C_{i}+C_{i-1}\right)\right]$$

At each new time step, additional ions, C_SRIM,_ are added into the bin for the next time step as well, leaving the final concentration at time t+Δt as,

(3) $$C_{i}\left(t+\Delta t\right)=C_{i}\left(t\right)+C_{\mathrm{SRIM},i}+\Delta C_{i}.$$

Note that these routines do not take radiation-induced segregation (RIS) into account and separate calculations must be made to estimate that effect. A full description of the input file needed for the code is given in the README.txt file.
